# Network pharmacology to explore the novel anti-inflammatory mechanism of naringenin in intestinal ischemia/reperfusion injury

**DOI:** 10.3389/fimmu.2025.1623080

**Published:** 2025-08-08

**Authors:** Min Hou, Yanshun Wang, Suheng Chen, Zhiguo Tan, Jie Liu, Xiaoxi Li, Xiaoxia Han, Zaiqi Yang, Yufang Leng

**Affiliations:** ^1^ The First School of Clinical Medicine, Lanzhou University, Lanzhou, China; ^2^ Department of Anesthesiology, Liaocheng Hospital of Traditional Chinese Medicine, Shandong, China; ^3^ Department of Anesthesiology, The Affiliated Taian City Central Hospital of Qingdao University, Taian, Shandong, China; ^4^ Department of Anesthesiology, The First Hospital of Lanzhou University, Lanzhou, China

**Keywords:** intestinal ischemia/reperfusion injury, network pharmacology, naringenin, inflammation, YAP, stat3

## Abstract

**Introduction:**

Naringenin (Nar), a common flavanone abundant in citrus fruits and tomatoes, is common in diets. Although Nar can alleviate intestinal ischemia/reperfusion injury (IRI), the exact anti-inflammatory mechanisms are unclear and require further study.

**Methods:**

In this study, we employed a comprehensive research strategy that integrated network pharmacology analysis with both *in vitro* and *in vivo* experimental validations to systematically elucidate Nar’s anti-inflammatory mechanisms in intestinal IRI.

**Results:**

Network pharmacology uncovered 88 common anti-inflammatory targets for Nar in intestinal IRI. Among these, TNF, IL6, AKT1, IL1B, TP53, STAT3, and PTGS2 were identified as hub genes. Validation experiments demonstrated that Nar induced anti-inflammatory responses through downregulating calprotectin, IL-1β, IL-6, and TNF-α, while promoting IL-10 secretion. Additionally, Nar pretreatment significantly downregulated PTGS2 and phosphorylated STAT3 (p-STAT3). Further mechanistic investigations were conducted using the YAP inhibitor verteporfin (VP) in vitro and in vivo. Nar pretreatment activated YAP, thereby enhancing its anti-inflammatory effects. Conversely, inhibiting YAP activation with VP increased p-STAT3 and enhanced inflammatory responses, diminishing Nar’s efficacy.

**Conclusion:**

This study demonstrated that Nar inhibited intestinal inflammatory responses by activating YAP, which suppressed p-STAT3 expression, and provided a theoretical basis for Nar’s clinical application in intestinal IRI.

## 1 Introduction

Intestinal ischemia/reperfusion injury (IRI) is a serious pathological course in the clinic characterized by high mortality, which may be present in a variety of clinical conditions, including cardiopulmonary bypass surgery, acute mesenteric ischemia, bowel transplants, bowel resections, abdominal aortic aneurysm surgery, and shock (1–3). It has been observed that intestinal IRI is associated with inflammation mediator release (4, 5). When pro-inflammatory and anti-inflammatory responses are imbalanced, the intestinal mucosa barrier becomes compromised, while mucosal permeability is increased (6, 7). Multiple signaling pathways are activated in this inflammatory response, causing inflammation storms that lead to multiple pathophysiological processes, including systemic inflammatory response syndrome (8). Numerous studies have recently demonstrated the contribution of inflammation to intestinal IRI (9). Therefore, inhibition of the inflammatory cascade is a fundamental therapeutic strategy for intestinal IRI.

Many advantages can be found in plant compounds, including low cost, multiple targets, low toxicity, and high availability, especially flavonoids, which offer numerous benefits to humans (10). Naringenin (4’,5,7-trihydroxyflavanone, Nar), found in grapefruits and sour oranges, is one of the most well-known flavonoids (11). Studies have shown that Nar exhibits anti-inflammatory and anti-infective effects (12–14). It was found that Nar alleviated retinal IRI by exerting anti-inflammatory effects (15). In diabetic rats and rats with ethanol-induced liver injury, Nar also showed excellent anti-inflammatory activity (16, 17). A study found that Naringin, one of Nar’s glycoside forms, reduced inflammation associated with intestinal IRI (18). Our previous study identified that Nar alleviated intestinal IRI (19). However, its precise anti-inflammatory mechanism remains to be elucidated. As a result, identifying potential therapeutic targets may help elucidate the anti-inflammatory mechanisms of Nar in intestinal IRI.

In recent years, bioinformatics approaches have helped systematically reveal the molecular mechanisms involved in complex diseases and drug actions (20). From a comprehensive and holistic perspective, network pharmacology can identify potential molecular targets and their fundamental mechanisms (21). This study, based on network pharmacology, identified common genes related to Nar, anti-inflammatory properties, and intestinal IRI, and aimed to predict how Nar regulates inflammation to alleviate intestinal IRI, and to provide insights into anti-IRI drugs.

## 2 Materials and methods

### 2.1 Network pharmacology and bioinformatics data collection

#### 2.1.1 Target prediction for Nar

With the CAS (480–41–1) of “Naringenin” as the key word, we identified the SMILES and structure of Nar from Pubchem (http://pubchem.ncbi.nlm.nih.gov/). Nar targets were searched through TCMSP (https://old.tcmsp-e.com/tcmsp.php/), CTD (https://ctdbase.org/), PharmMapper (http://www.lilab-ecust.cn/pharmmapper/), Similarity Ensemble Approach (SEA, https://sea.bkslab.org/), and SwissTargetPrediction (http://www.swisstargetprediction.ch/).

#### 2.1.2 Targeting intestinal IRI and inflammation-related targets

Intestinal IRI targets were gathered using keywords “intestinal ischemia reperfusion injury” from Online Mendelian Inheritance in Man (OMIM, http://omim.org) (22), PharmGKB (https://www.pharmgkb.org/), as well as Gene Cards (http://www.genecards.org) databases (23). We selected genes that scored above 1 as potential targets from the Gene Cards database. Inflammation-related targets were acquired from OMIM and Gene Cards with “anti-inflammatory”.

#### 2.1.3 Identification of core target genes

The online platform Venny 2.1.0 (https://bioinfogp.cnb.csic.es/tools/venny/) was utilized to identify shared genes between Nar targets, intestinal IRI targets, and anti-inflammatory targets. These intersecting genes were potential key targets for Nar-mediated anti-inflammatory mechanisms in intestinal IRI.

#### 2.1.4 Protein protein interactions (PPI) networks

We created PPI networks using STRING (https://cn.string-db.org/) based on the identified common genes and visualized them using Cytoscape 3.7.2. We set confidence scores above 0.7. Based on CytoHubba, more therapeutic targets were screened (24).

#### 2.1.5 Enrichment analysis of gene ontology (GO) and kyoto encyclopedia of genes and genomes (KEGG)

GO functional and KEGG pathway analyses were gathered from DAVID (https://david.ncifcrf.gov) (25). Visualization of the enrichment results was performed on bioinformatics.com.cn.

#### 2.1.6 Molecular docking

We used AutoDockTools 1.5.6 software for flexible docking of Nar and candidate targets to assess Nar’s binding affinity. PDB and PubChem databases were used to download drug ligands and protein receptors (26). Pymol 2.2.0 was used to visualize the proteins with the lowest scores.

### 2.2 Experimental verification

#### 2.2.1 Antibodies and chemicals

Nar (M4000, purity >98.5%) were purchased from AbMole (Shanghai, China). Verteporfin (VP, HY-B0146) and hypoxia-inducible factor (HIF)-1α (HY-P80704) were purchased from MedChemExpress (NJ, USA). Occludin (PA6013) and tumor necrosis factor (TNF)-α (PY19810) were purchased from Abmart (Shanghai, China). Zonula occluden-1 (ZO-1, GTX114949) was obtained from GeneTex. 705-phosphorylated signal transducer and activator of transcription 3 (p-STAT3, ET1603-40) and STAT3 (SY24-08) were obtained from Huabio (Zhejiang, China). Cyclooxygenase 2 (COX2; PTGS2, 12375-1-AP), Yes-associated protein (YAP, 13584-1-AP), Interleukin (IL)-1β (26048-1-AP) and Lamin B1 (12987-1-AP) were obtained from Proteintech Group (Wuhan, China). IL-6 (WL02841) and IL-10 (WL03088) were obtained from Wanleibio (Shenyang, China). The anti-β-actin antibody (BM3873) was provided by BOSTER (China). Nuclear protein extraction kit (EX1470) was purchased from Jiancheng (Nanjing, China).

#### 2.2.2 Animals and cells

Male C57BL/6 J mice (6–8 weeks old) were purchased from Lanzhou veterinary research institute (Certificate of Conformity: No. SCXK (Gan) 2020-0002). The protocol was approved by the Animal Protection and Ethics Committee (Approval No.: LDYYLL2024-314). We followed internationally accepted principles for using and caring for animals in this study. All mice were housed for 7 days in SPF-rated rooms during the experiment.

IEC-6 cells were cultured with 10% FBS and 1% penicillin/streptomycin in DMEM medium. The cells were cultured in an incubator under a humidified atmosphere of 5% CO_2_ and 95% air at 37°C.

#### 2.2.3 Establishment of intestinal IRI models *in vivo* and *in vitro*

The mouse model of intestinal IRI has been described previously (19). Briefly, mice were given pentobarbital (30 mg/kg) intraperitoneally after fasting for 12 h (free of water). After 45 minutes of ischemia, the superior mesenteric artery (SMA) was reperfused for another 30 minutes.

The oxygen-glucose deprivation/reoxygenation (OGD/R) model was established as previously reported (19). Briefly, IEC-6 cells were exposed to hypoxia under anaerobic conditions (5% CO_2_ and 95% N_2_) in PBS for 3 h, followed by reoxygenation under normoxic conditions for 1 h.

#### 2.2.4 Experimental design

Nar’s effect on intestinal IRI was investigated *in vivo* by randomly dividing mice into 5 groups: Sham group (0.5% sodium carboxymethylcellulose, CMC-Na), Sham+Nar (H) group (high doses of Nar, 100 mg/kg), IR group, IR+Nar (L) group (low doses of Nar, 50 mg/kg), and IR+Nar (H) group (high doses of Nar, 100 mg/kg). The mice were received CMC-Na or Nar daily for 7 days before intestinal IRI.

Nar’s effect on OGD/R injury was investigated *in vitro* by randomly dividing IEC-6 cells into 4 groups: Control (Con) group: the cells were cultured in normal medium, Con+Nar group, OGD/R group, and OGD/R+Nar group. Before OGD/R, Nar (75 μM) was administered for 12 h to the cells.

To examine how YAP contributed to Nar’s anti-inflammatory effects *in vivo*, mice were randomly divided into 4 groups: Sham group, IR group, IR+Nar group, and IR+Nar+VP group. In order to inhibit YAP, VP (100 mg/kg) was administered intraperitoneally prior to surgery.

To examine how YAP contributed to Nar’s anti-inflammatory effects *in vitro*, we divided IEC-6 cells into 4 groups: Con group, OGD/R group, OGD/R+Nar group, and OGD/R+Nar+VP group. We added VP (1 μM) 12 h before OGD/R.

#### 2.2.5 Histological analysis

At the end of the reperfusion, we anesthetized the mice and collected intestinal tissues, fixed them in 4% paraformaldehyde, and stained them with hematoxylin-eosin (H&E) and immunohistochemistry (IHC) staining. Using Chiu’s score, a double-blind method was used to assess intestinal histopathological lesions (27).

#### 2.2.6 Enzyme-linked immunosorbent assay (ELISA)

Intestinal tissue and serum levels of IL-6, IL-1β, IL-10 and TNF-α were determined using ELISA kits (RUIXIN, Fujian, China). Meanwhile, the intestinal tissue level of calprotectin was determined using an ELISA kit (MM-1180M2, Meimian, Jiangsu, China).

#### 2.2.7 Western blotting (WB) assay

Protein samples from bowel tissue or cells collected following reperfusion were prepared using RIPA lysis buffer supplemented with the protease inhibitor cocktail (EpiZyme, Cat No. GRF101). A nuclear/cytoplasmic fractionation kit was used to extract nuclear protein according to the instructions. The membranes were blocked with 5% non-fat milk for 1 h and then incubated at 4°C overnight with the primary antibodies: ZO-1 (1:1000), Occludin (1:5000), p-STAT3 (1:2000), STAT3 (1:2000), IL-6 (1:1000), TNF-α (1:1000), IL-10 (1:1000), IL-1β (1:1000), PTGS2 (1:2000), YAP (1:8000), HIF-1α (1:500), β-actin (1:10000), Lamin B1 (1:20000). Subsequently, membranes were exposed to appropriate secondary antibodies for 1 h, and proteins were visualized with enhanced chemiluminescence (Sparkjade Company, China). Semi-quantitative densitometric analyses were performed using Image J.

#### 2.2.8 Immunofluorescence staining

IEC-6 cells were treated with 4% paraformaldehyde and 5% BSA solution. The cells were incubated at 4°C overnight with anti-ZO-1, anti-Occludin, anti-STAT3, and anti-YAP antibodies. Meanwhile, intestinal tissue sections were incubated at 4°C overnight with anti-YAP antibodies. After being incubated with FITC-conjugated secondary antibodies and DAPI, cells and sections were imaged with fluorescent microscopy (Leica, Germany).

#### 2.2.9 Quantitative real-time polymerase chain reaction assay (RT-qPCR)

RT-qPCR was performed according to the previous description (28). **Table 1** presented the entire primer sequences synthesized by Sangon Biotech (Shanghai, China) for IEC-6 cells.

**Table 1:** The whole primer sequences.

Target	Sequences
Rat IL-6-F	5’-ACTTCCAGCCAGTTGCCTTCTTG-3’
Rat IL-6-R	5’-TGGTCTGTTGTGGGTGGTATCCTC-3’
Rat TNF-F	5’-CACCACGCTCTTCTGTCTACTGAAC-3’
Rat TNF-R	5’-TGGGCTACGGGCTTGTCACTC-3’
Rat β-actin-F	5’-GCTGTGCTATGTTGCCCTAGACTTC-3’
Rat β-actin -R	5’-GGAACCGCTCATTGCCGATAGTG-3’

### 2.3 Statistical analysis

GraphPad Prism 9.0 was performed for all statistical analyses. The data are presented as mean ± standard deviation (SD). We compared the groups using a one-way analysis of variance (ANOVA) followed by Tukey’s *post-hoc* test. *P*<0.05 was considered statistically significant.

## 3 Results

### 3.1 Targets identification

**Figure 1:**
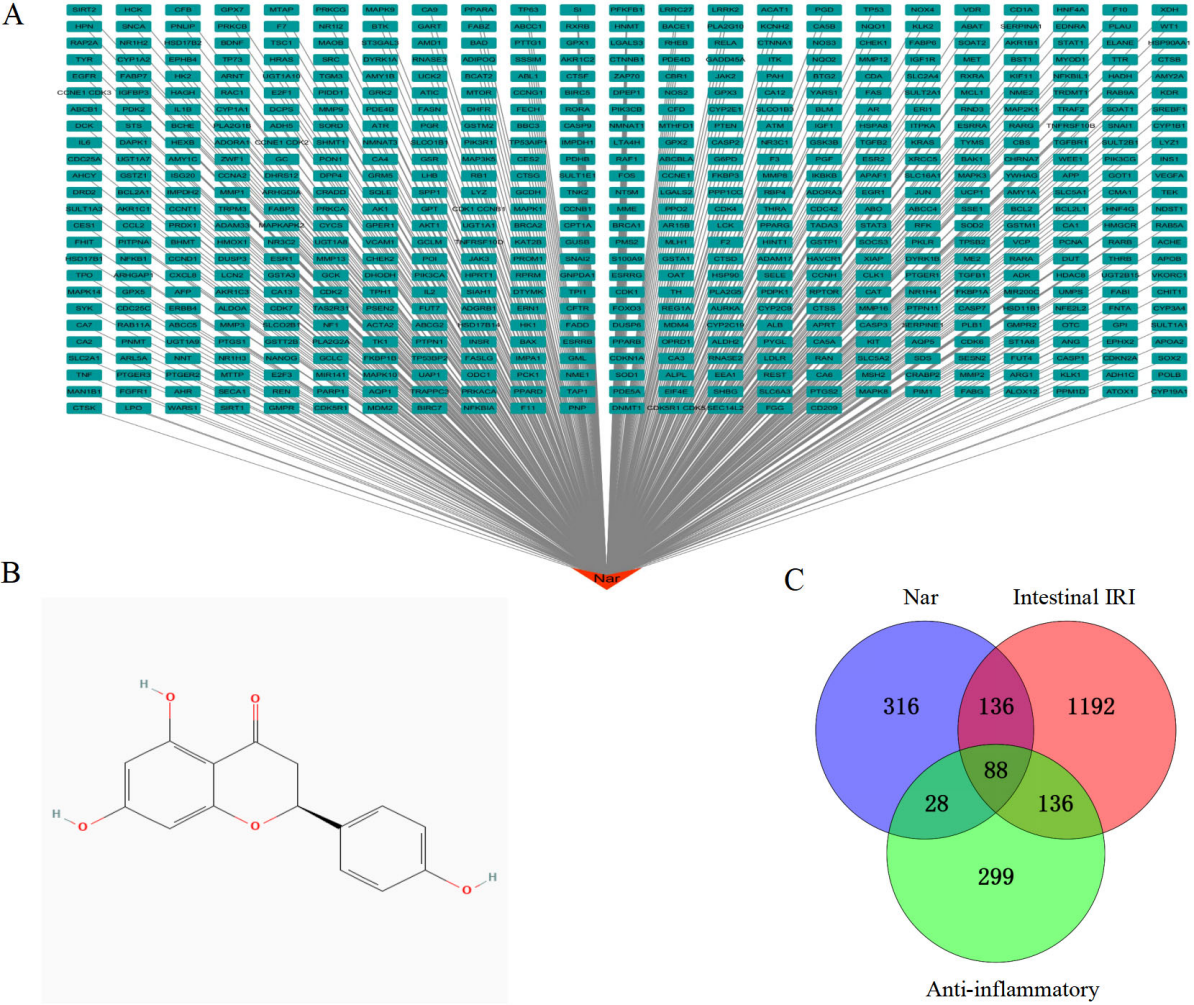
Identification of targets. **(A)** Network of drug targets: Red is Nar, green are potential candidates for Nar. **(B)** The chemical structure of Nar. **(C)** A Venn diagram illustrates shared targets in Nar, intestinal IRI and anti-inflammatory.

Using the Puchem database, we obtained Nar’s 2D structure (**Figure 1B**). With the limitation of “human species”, targets for Nar were obtained from 5 open-source databases, TCMSP (37), CTD (218), Pharmmapper (293), SEA (53), and SwissTargetPrediction (100). After eliminating duplicates, 568 targets related to Nar were acquired. We obtained network diagrams for Nar targets using Cytoscape 3.7.1 software, as shown in **Figure 1A**. We screened 1552 intestinal IRI genes in OMIM, Gene Cards, and PharmGKB. Genecards (relevance scores >5) and OMIM databases were used to identify 551 intersectional anti-inflammatory targets. With the duplicates removed, 88 intersection targets in the Venn diagram were generated by merging 568 Nar genes, 551 anti-inflammatory targets, and 1552 intestinal IRI genes (**Figure 1C**).

### 3.2 PPI network construction and hub gene identification

**Figure 2:**
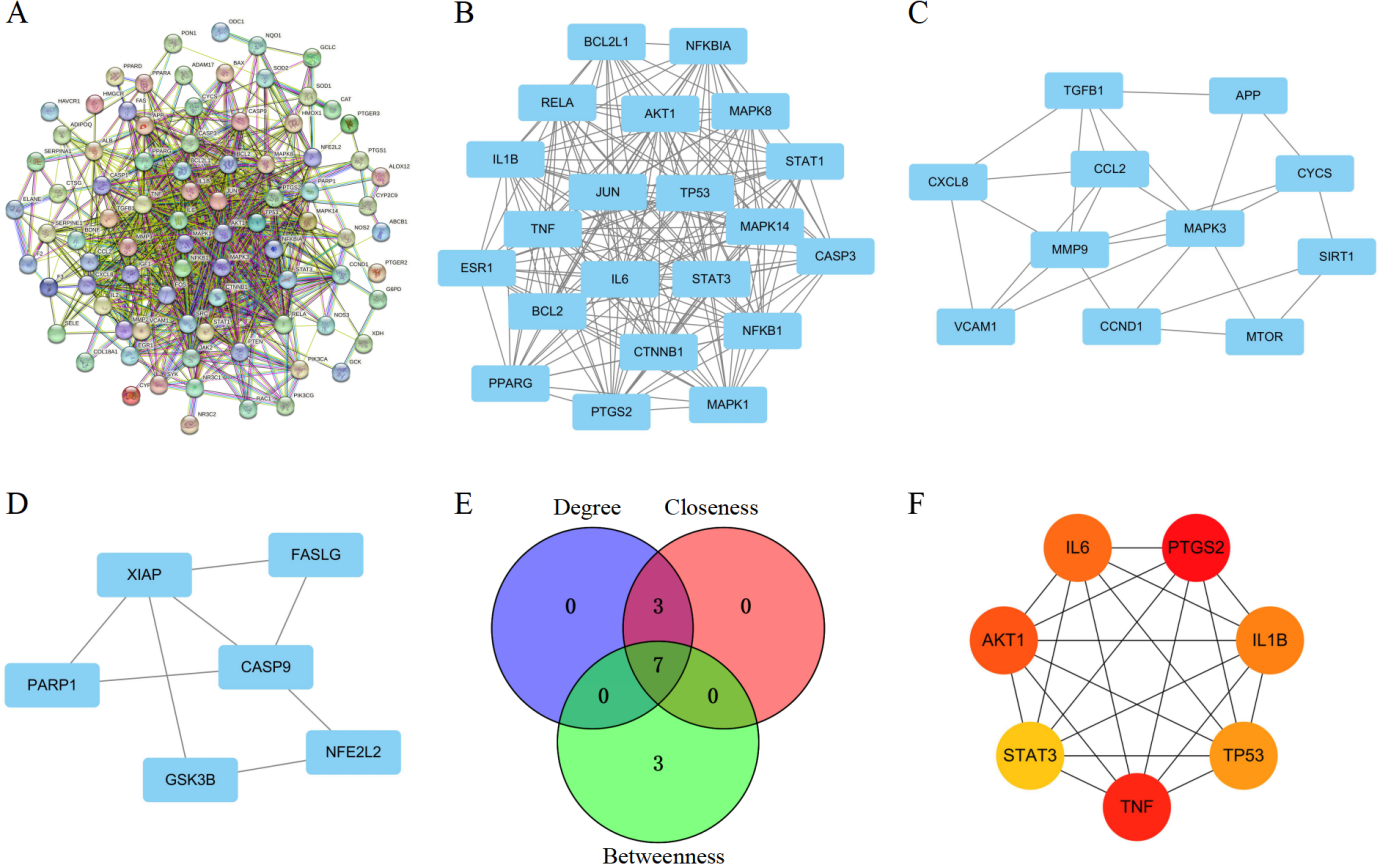
Identification of PPI network and core targets. **(A)** PPI network for hub targets of Nar-mediated anti-inflammatory in intestinal IRI. **(B–D)** The significant densely-connected modules identified by MCODE plug-in. **(E)** A Venn diagram illustrates shared targets using Closeness, Degree, and Betweenness. **(F)** A topological analysis of 7 hub genes in the PPI network.

To determine potential target genes interactions, a PPI network was screened by STRING. This network included 88 nodes and 737 edges, and *P* value < 1.0e-16 (**Figure 2A**). To identify critical clustering modules, the Cytoscape plug-in MCODE was applied. We retrieved three modules from the PPI network constructed using common genes. As shown in **Figure 2B**, Module 1 contained 21 nodes and 187 edges (cluster score: 18.7). Module 2, with 11 nodes and 25 edges (cluster score: 5.0) (**Figure 2C**). Module 3, with 6 nodes and 8 edges (cluster score: 3.2) (**Figure 2D**). CytoHubba was used to identify hub genes. Our prediction and exploration of the top 10 hub genes in the PPI networks was based on Closeness (TNF, AKT1, IL6, IL1B, TP53, STAT3, BCL2, JUN, PTGS2, CASP3), Degree (TNF, IL6, AKT1, IL1B, TP53, STAT3, JUN, BCL2, PTGS2, CASP3), and Betweenness (PTGS2, TNF, ALB, AKT1, IL6, IL1B, TP53, MMP9, STAT3, SRC). Based on the intersection of these 10 genes from the three algorithms, 7 candidates were identified as hub genes: TNF, IL6, AKT1, IL1B, TP53, STAT3, and PTGS2 (**Figures 2E, F**).

### 3.3 Enrichment analysis and molecular docking

**Figure 3:**
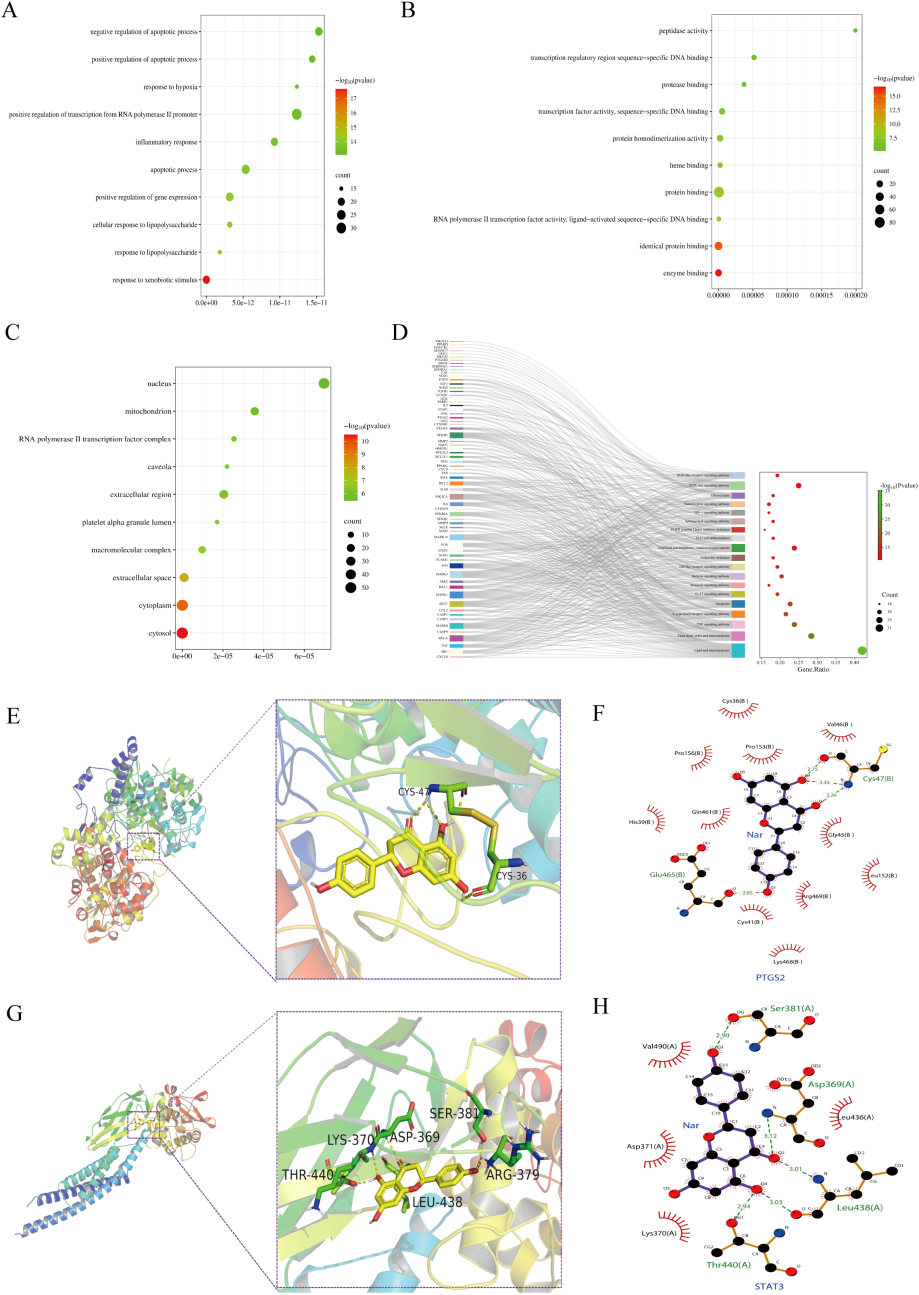
Enrichment analysis and molecular docking. **(A–C)** The enrihment analysis of the BP **(A)**, MF **(B)**, and CC **(C)** modules. **(D)** KEGG pathway related genes: Sankey diagram of topically enriched terms. **(E, F)** PTGS2 binding mode in three dimensions and two dimensions (PDB: 5F19). **(G, H)** STAT3 binding mode in three dimensions and two dimensions (PDB: 6NJS).

767 significant items were found in the GO functional annotations (**Figures 3A–C**). Among these, 606 biological processes (BP) were found (**Figure 3A**), mainly involved in inflammatory response, response to hypoxia, and gene expression regulation; A total of 98 molecular functions (MF) were identified for enzyme binding, protein binding, heme binding, sequence-specific DNA binding etc. (**Figure 3B**); and 63 cellular components (CC) terms were found for cytosol, cytoplasm, nucleus, mitochondrion, extracellular space etc. (**Figure 3C**). Based on the KEGG pathway analysis, 161 pathways were identified (**Figure 3D**), including pathways for TNF, IL-17, Toll-like receptor, HIF-1, and PI3K-Akt. These analyses highlighted possible molecular mechanisms whereby Nar reduces intestinal IRI.

Based on the molecular docking results, Nar showed high binding affinity towards all targets tested, with binding free energies < -5.0 kcal/mol (STAT3: -7.2 kcal/mol, PTGS2: -9.5 kcal/mol). Nar formed hydrogen bonds with STAT3 at LYS-370, THR-440, ASP-369, SER-381, LEU-438, ARG-379, and with PTGS2 at CYS-36, CYS-47 (**Figures 3E–H**).

### 3.4 Nar attenuated intestinal IRI *in vivo* and *in vitro*

**Figure 4:**
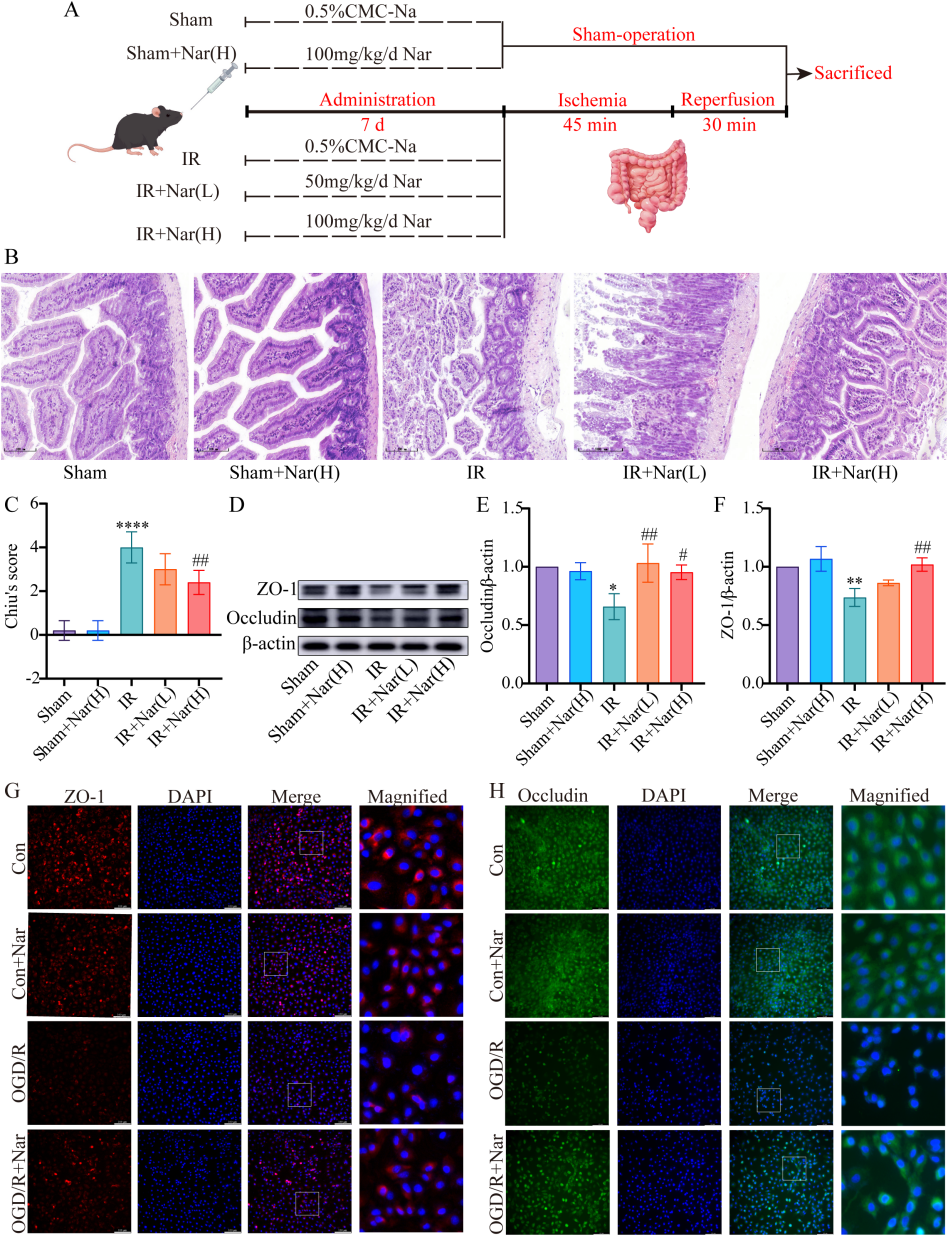
Nar protected against intestinal IRI *in vivo* and *in vitro*. **(A)** The diagram of experimental design by FigDraw. **(B)** H&E staining (magnification ×200, scale = 100 μm). **(C)** Chiu’s score of intestinal tissues. **(D–F)** The relative protein expression of ZO-1 and Occludin in intestinal tissues. **(G, H)** Representative immunofluorescence staining of ZO-1 and Occludin in IEC-6 cells (scale bar = 100 μm; magnification ×200). (*n*=3-5). ^*^ *P* < 0.05, ^**^ *P* < 0.01, ^****^ *P* < 0.0001 versus Sham group; ^#^ *P* < 0.05, ^##^ *P* < 0.01 versus IR group.

To investigate the role of Nar in intestinal IRI, a murine intestinal IRI model was established and treated with Nar (50 mg/kg, 100 mg/kg) for 7 days (**Figure 4A**). H&E staining as well as tight junctions (TJs) levels were used to investigate whether Nar protected against intestinal IRI in mice. Compared to sham mice and Nar-treated sham mice, an increase in Chiu’s score was observed in the IR group with distorted arrangement of vili, extensive destruction of the intestinal epithelium, and discontinuous arrangement in the intestinal epithelium (**Figures 4B, C**). Meanwhile, the WB revealed decreased expressions of ZO-1 and Occludin in the IR group (**Figures 4D–F**). Pretreatment with Nar (100 mg/kg) greatly reduced the IRI-induced intestinal mucosa injury by restoring Chiu’s score and increasing ZO-1 and Occludin (**Figures 4D–F**).

*In vitro*, Occludin and ZO-1 expression levels were assessed by immunofluorescence analysis. There was a significant reduction in fluorescence intensity and regularity in the OGD/R group versus the Con group, suggesting compromised TJs. However, Nar pretreatment restored fluorescence intensity, suggesting TJs were restored (**Figures 4G, H**). These results suggested that Nar effectively reduced IRI-induced intestinal epithelial damage.

### 3.5 Nar reduced inflammation induced by intestinal IRI *in vivo* and *in vitro*

**Figure 5:**
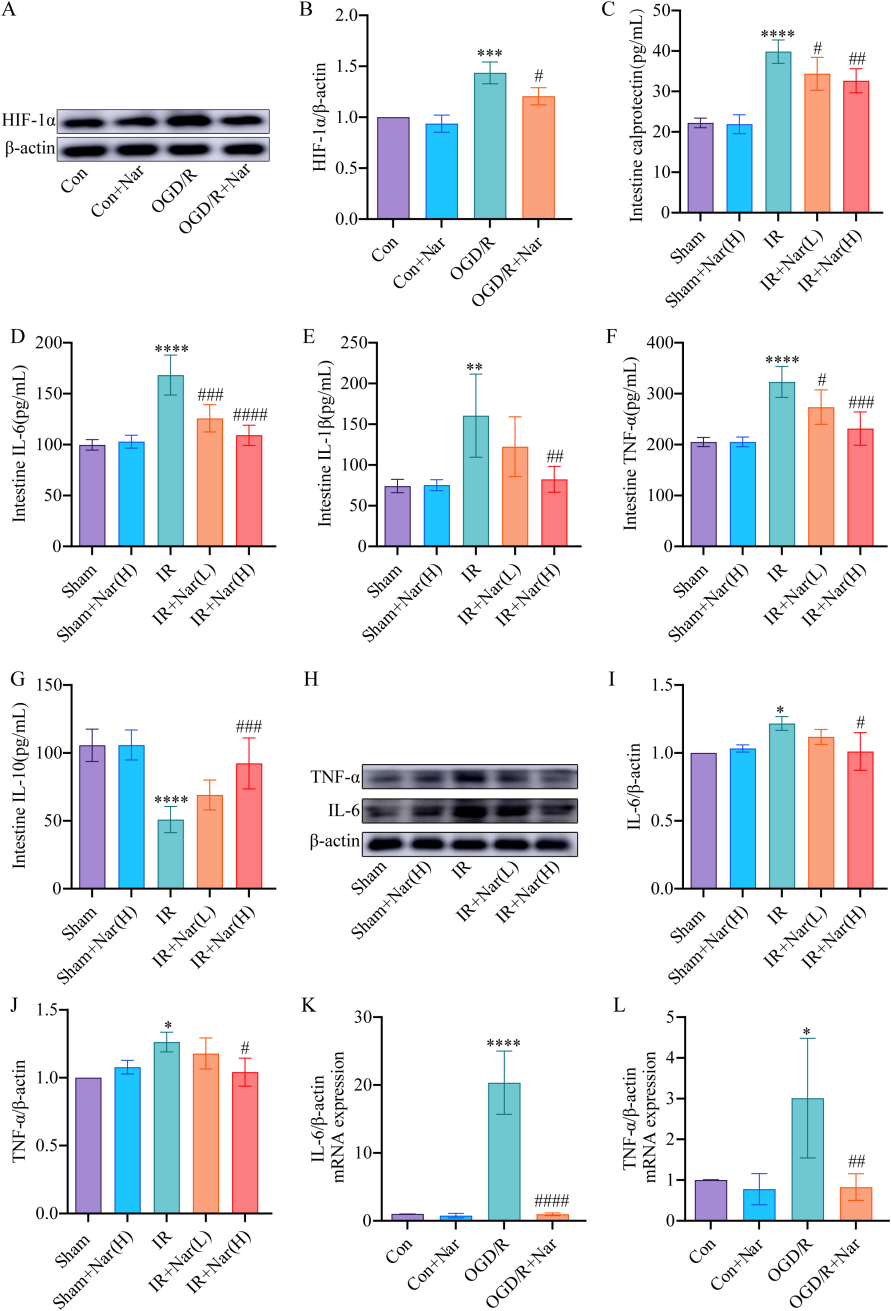
Nar pretreatment attenuated inflammation induced by intestinal IRI *in vivo* and *in vitro*. **(A, B)** The relative level of HIF-1α in IEC-6 cells. **(C–G)** Calprotectin, IL-6, IL-1β, TNF-α, and IL-10 levels of intestinal tissues. **(H–J)** The relative protein expression of TNF-α and IL-6 in intestinal tissues. **(K, L)** The mRNA levels of IL-6 and TNF-α in IEC-6 cells. (*n*=3-5). ^*^ *P* < 0.05, ^**^ *P* < 0.01, ***P < 0.001, ^****^ *P* < 0.0001 versus Sham (Con) group; ^#^ *P* < 0.05, ^##^ *P* < 0.01, ^###^ *P* < 0.001, ^####^ *P* < 0.0001 versus IR (OGD/R) group.

HIF-1α is a transcription factor that is expressed in hypoxic conditions. We first evaluated Nar’s effect on HIF-1α in OGD/R-induced IEC-6 cells. WB revealed increased expression of HIF-1α in OGD/R group (**Figures 5A, B**). And the level of HIF-1α was significantly recovered by Nar pretreatment *in vitro* (**Figures 5A, B**). Calprotectin and inflammatory cytokines (TNF-α, IL-6, IL-1β, and IL-10) were assessed in mouse colon tissue. ELISA results demonstrated that in the intestinal IRI models, calprotectin, TNF-α, IL-1β, and IL-6 elevated, whereas IL-10 was reduced. In contrast, Nar pretreatment reduced calprotectin, TNF-α, IL-6, and IL-1β, and increased IL-10 compared with IR group (**Figures 5C–G**). We also detected TNF-α and IL-6 levels by WB. In comparison with the Sham group, the IR group showed higher levels of TNF-α and IL-6. Compared with the IR group, TNF-α and IL-6 expression was lower in the IR+Nar (H) group (**Figures 5H–J**). *In vitro*, we analyzed TNF-α and IL-6 levels in IEC-6 cells using RT-qPCR techniques. In response to OGD/R, TNF-α and IL-6 mRNA levels were upregulated compared with the Con group. Importantly, the administration of Nar in the OGD/R+Nar group significantly reduced TNF-α and IL-6 mRNA expression compared with the OGD/R group (**Figures 5K, L**). Our results supported the hypothesis that Nar pretreatment inhibited intestinal IRI- induced inflammatory response.

3.6 Nar regulated PTGS2 and STAT3 expression in intestinal IRI *in vivo* and *in vitro*

**Figure 6:**
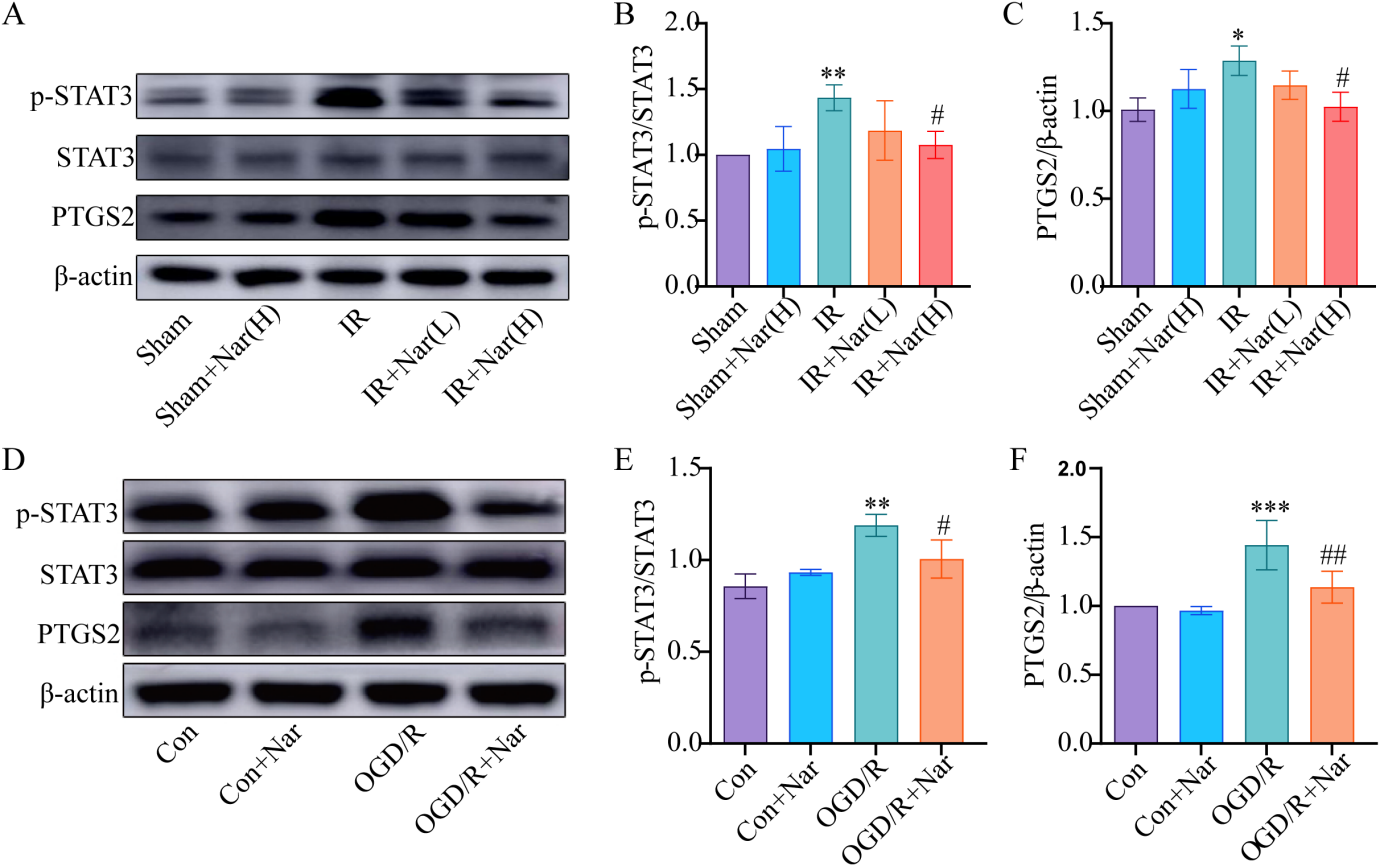
Effects of Nar on STAT3 and PTGS2 in intestinal IRI *in vivo* and *in vitro*. **(A–C)** The relative levels of STAT3 and PTGS2 in intestinal tissues; **(D–F)** The relative levels of STAT3 and PTGS2 in IEC-6 cells. (*n*=3-5). ^*^ *P* < 0.05, ^**^ *P* < 0.01, ^***^ *P* < 0.001 versus Sham (Con) group; ^#^ *P* < 0.05, ^##^ *P* < 0.01, versus IR (OGD/R) group.

To further validate the analysis of the PPI network and molecular docking, we further assessed the expression of PTGS2 and STAT3. WB revealed that PTGS2 and p-STAT3 expressions were significantly increased in IR group compared with the Sham group. Nar pretreatment significantly downregulated PTGS2 and p-STAT3 expression in the IR+Nar group (**Figures 6A–C**). Similarly, OGD/R induced upregulation of PTGS2 and p-STAT3 in IEC-6 cells, while Nar pretreatment downregulated PTGS2 and p-STAT3 levels in the OGD/R+Nar group (**Figures 6D–F**). Therefore, we speculated that Nar inhibited inflammation by reducing PTGS2 and p-STAT3 protein expression.

3.7 Nar inhibited inflammatory response by activating YAP *in vivo* and *in vitro*

**Figure 7:**
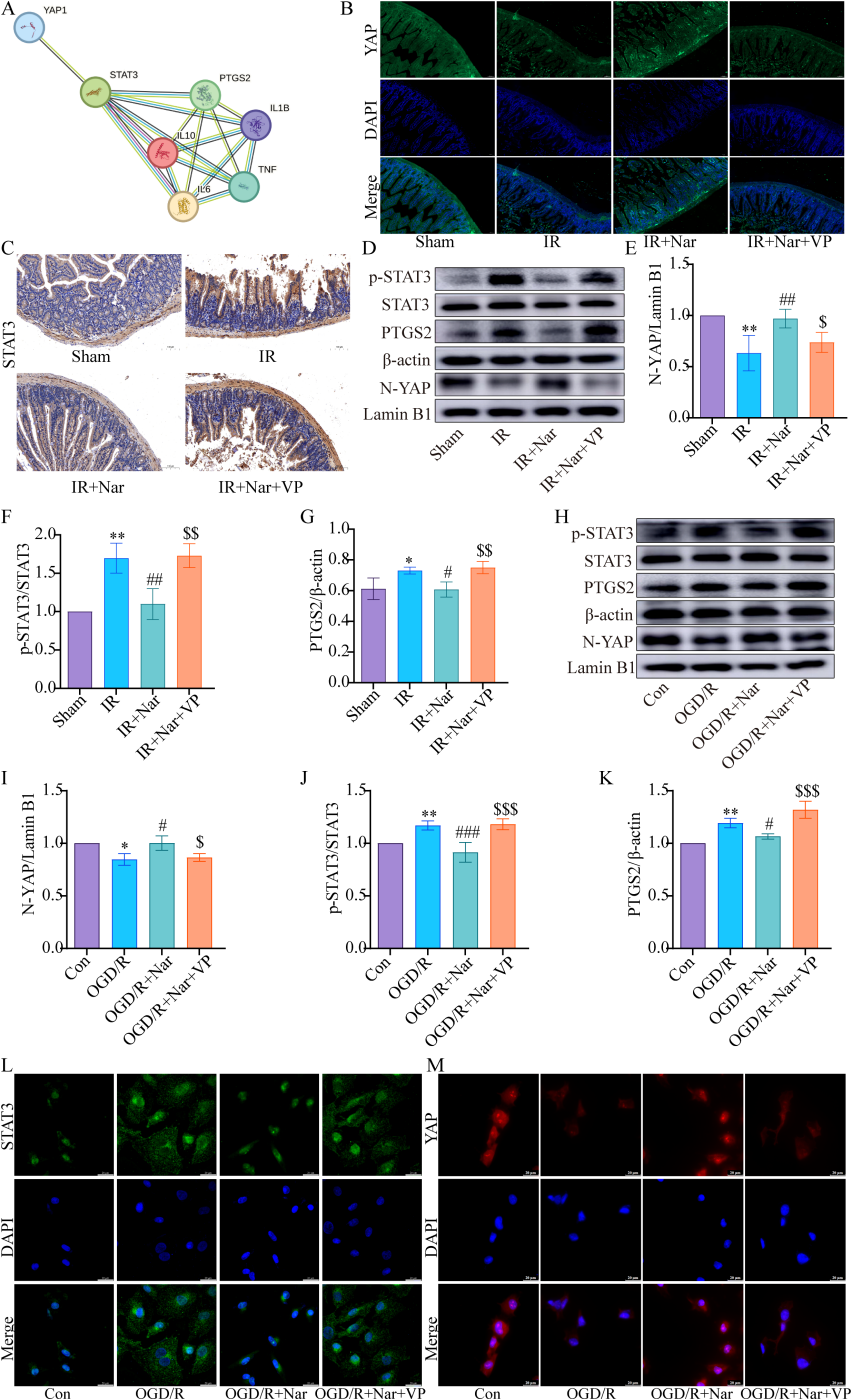
Nar inhibited p-STAT3 and PTGS2 levels by activating YAP expression *in vivo* and *in vitro*. **(A)** The PPI network downloaded from STRING database indicted the interaction between YAP, STAT3, PTGS2, IL6, TNF, IL1B, and IL10. **(B)** Representative immunofluorescence staining images of YAP in intestinal tissues (scale bar = 50 µm; magnification × 200). **(C)** IHC staining of STAT3 in intestinal tissues (scale bar = 100 μm; magnification × 200). **(D–G)** The relative levels of YAP, STAT3 and PTGS2 in intestinal tissues; **(H–K)** The relative levels of YAP, STAT3 and PTGS2 in IEC-6 cells. **(L, M)** Representative immunofluorescence staining images of STAT3 and YAP in IEC-6 cells. (scale bar = 20 µm; magnification × 1000). (*n*=3-5). ^*^ *P* < 0.05, ^**^ *P* < 0.01 versus Sham (Con) group; ^#^ *P* < 0.05, ^##^ *P* < 0.01, ^###^ *P* < 0.001 versus IR (OGD/R) group; ^$^ *P* < 0.05,^$$^ *P* < 0.01, ^$$$^ *P* < 0.001 versus IR+Nar (OGD/R+Nar) group.

Our previous studies confirmed that Nar inhibited ferroptosis by activating YAP in intestinal IRI (19). Based on STRING database prediction, we found an interaction between YAP, STAT3, PTGS2, IL6, TNF, IL1B, and IL10 (**Figure 7A**). Based on our previous Co-IP results, YAP interacted with STAT3 (19). For the study of the YAP/STAT3 axis in inflammation, mice were treated with VP against YAP expression. WB and immunofluorescence analysis revealed reduced nuclear translocation of YAP in IR group; Nar pretreatment activated YAP and increased YAP nuclear translocation level in IR+Nar group; With VP applied, YAP nuclear translocation levels decreased significantly in the IR+Nar+VP group (**Figure 7B, D, E**). Meanwhile, Nar pretreatment reversed intestinal IRI-induced upregulation of p-STAT3 and PTGS2 (**Figure 7D, F, G**); With VP applied, p-STAT3 and PTGS2 levels increased significantly in the IR+Nar+VP group (**Figures 7D, F, G**). IHC results of STAT3 were consistent with WB (**Figure 7C**). Similar results were also observed *in vitro*. After inhibiting YAP expression by VP, Nar failed to activate YAP (**Figures 7H, I**). In the OGD/R+Nar+VP group, with VP applied, the p-STAT3 and PTGS2 levels increased significantly (**Figures 7H, J, K**). Immunofluorescence results also supported that Nar significantly up-regulated YAP, while down-regulating STAT3 in IEC-6 cells, but VP reversed the above indicators (YAP, STAT3) in the OGD/R+Nar+VP group (**Figures 7L, M**).

**Figure 8:**
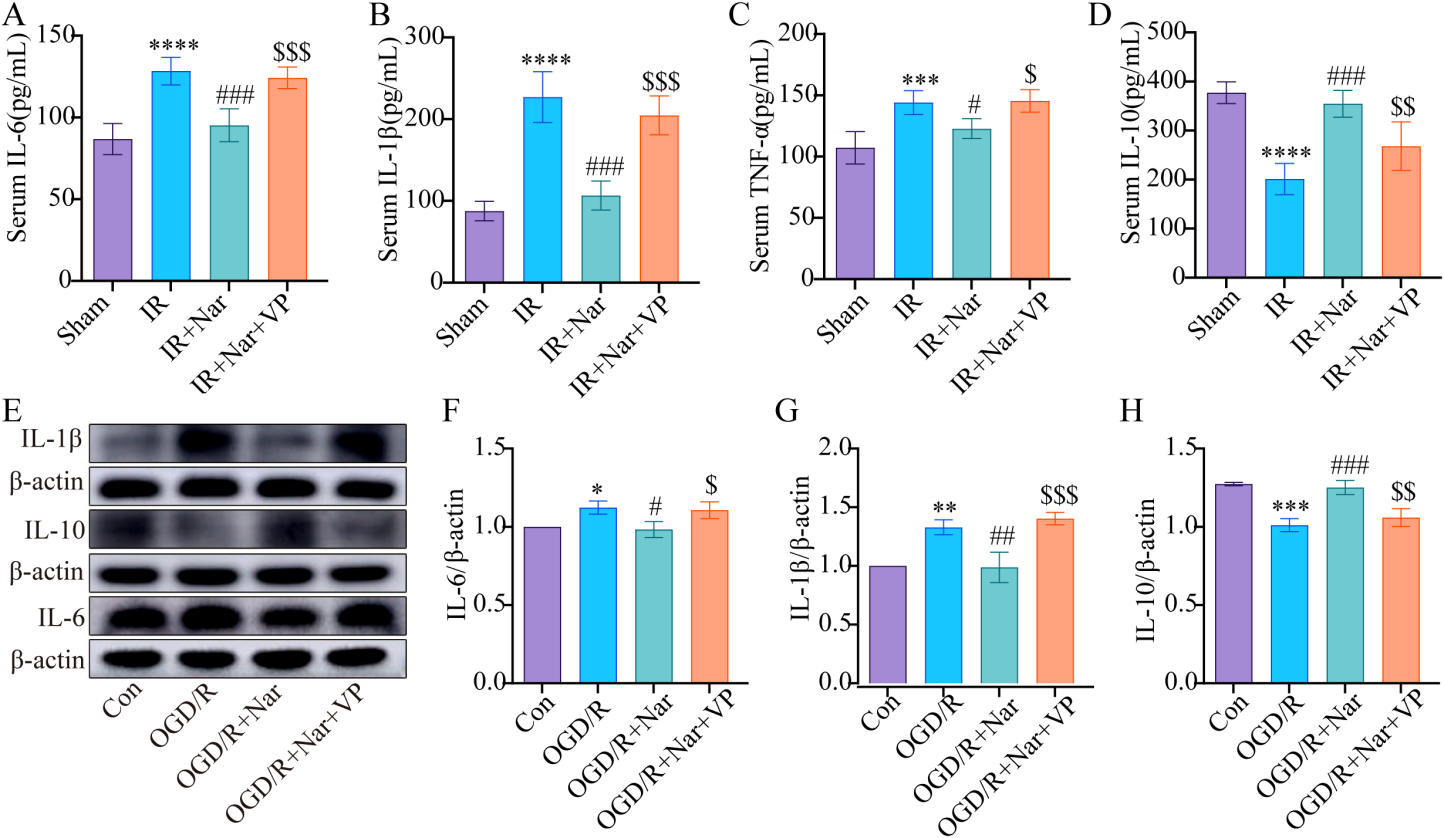
Nar inhibited inflammatory response by activating YAP expression *in vivo* and vitro. **(A–D)** IL-6, IL-1β, TNF-α, and IL-10 levels in serum. **(E–H)** The relative levels of IL-1β, IL-10 and IL-6 in IEC-6 cells. (*n*=3-5). ^*^ *P* < 0.05, ^**^ *P* < 0.01, ^***^ *P* < 0.001 ^****^ *P* < 0.0001 versus Sham (Con) group; ^#^ *P* < 0.05, ^##^ *P* < 0.01, ^###^ *P* < 0.001 versus IR (OGD/R) group; ^$^ *P* < 0.05, ^$$^ *P* < 0.01, ^$$$^ *P* < 0.001 versus IR+Nar (OGD/R+Nar) group.

In addition, we examined how VP affected inflammation. *In vivo*, ELISA analysis showed that Nar had no effect on reducing TNF-α, IL-1β, and IL-6, while increasing IL-10 in the IR+Nar+VP group (**Figures 8A–D**). In the OGD/R model, WB analysis revealed that Nar significantly reduced IL-6 and IL-1β while increasing IL-10 expression (**Figures 8E–H**). However, the addition of VP reversed these effects, leading to a significant decrease in IL-10 expression and a corresponding increase in IL-6 and IL-1β in OGD/R+Nar+VP group (**Figures 8E–H**). In conclusion, Nar suppressed STAT3 phosphorylation by promoting YAP expression, which alleviated intestinal IRI-induced inflammation.

## 4 Discussion

Intestinal IRI is a life-threatening disease in the clinical setting. It is strongly associated with the onset, progression, and prognosis of various clinical illnesses (29–31). If not diagnosed and treated promptly, acute mesenteric ischemia mortality can reach 60%-80% (32). At present, there is no clear treatment for intestinal IRI. It is well known that Nar, which is a flavanone found in citrus fruits and tomatoes, is crucial to the human diet. As a result of its extensive anti-diabetic, anti-atherogenic, anti-inflammatory, immunomodulatory, and antioxidative properties, it has gained increasing attention (33). Many studies have shown that Nar can mitigate IRI in various organs, especially the heart, brain, and kidneys (34–36). Our previous studies demonstrated that Nar relieved intestinal IRI by inhibiting ferroptosis (19). In this study, our network pharmacology analysis combined with experimental validation indicated that Nar was beneficial for intestinal IRI-induced inflammation.

Inflammation induced by intestinal IRI damages the intestinal barrier. Our data indicated the protective effects of Nar treatment against intestinal IRI-induced gut barrier injury via upregulation of Occludin and ZO-1. A recent study showed Nar protected the colonic mucosal layer in inflammatory bowel disease (IBD) (37, 38). Nar reduced radiation-induced intestinal permeability and dysfunction (39). And these findings were consistent with our results, proving that Nar protected against intestinal IRI. Nar has a promotional effect on barrier integrity (40). In two studies using Caco-2 cell monolayers, Nar improved intestinal barrier function, as shown by higher expression levels of TJs (41, 42). However, in Nar-treated sham mice, the levels of Occludin and ZO-1 was not significant increase in our study. These results suggest that Nar selectively improves the intestinal barrier and appear to be highly dependent on the type of species (animals, cells), Nar treatment (dose, method, timing), and detection method used.

Inflammation is a ubiquitous physiological response, acting as a defensive mechanism against a variety of injurious stimuli (43). Over-activation of inflammation cells and release of cytokines can severely damage intestinal epithelia (44, 45). An imbalance between pro-inflammatory and anti-inflammatory factors, as the chief contributor, can exacerbate intestinal IRI (46–48). Calprotectin is a cytoplasmic protein mostly produced by neutrophils, which is released following cell death (49). As pro-inflammatory protein, calprotectin is an effective marker for acute and chronic inflammation. In our study, calprotectin, IL-1β, IL-6, and TNF-α increased, while IL-10 decreased under IRI and OGD/R conditions. Nar pretreatment decreased proinflammatory factors expression and increased IL-10 levels. As shown in previous studies, Nar protected against IBD by inhibiting the inflammatory cascade (37, 38). In addition, Nar inhibited radiation-induced lung injury by downregulating IL-1β and restoring inflammatory factor homeostasis (50). In cerebral IRI, Nar prevented OGD/R- or IRI-induced inflammatory injury (51, 52). Our findings support these findings, showing Nar could protect against intestinal IRI by inhibiting inflammation.

STAT3 is a cytoplasmic transcription factor that transmits extracellular cytokine and growth factor signals and activates gene expression (53). It is well documented that STAT3 signaling is activated during intestinal IRI, and it is deeply connected to inflammation, oxidative stress, and apoptosis (54). When inflammatory cytokines, like IL-6, are bound to their receptors, the signal transducer gp130 is activated, leading to STAT3 activation (55). After that, phosphorylated STAT3 dimers translocate to the nucleus and initiate gene transcription (56). It was found that inhibiting STAT3 signaling activation reduces intestinal injury, by inhibiting apoptosis and the inflammatory response evoked by IRI (57, 58). Consequently, STAT3 could be a crucial target for intestinal IRI by controlling the inflammatory response. Consistent with the present study, p-STAT3 was significantly higher in the IR group, while Nar reduced p-STAT3, indicating that Nar could improve intestinal IRI by inhibiting p-STAT3 levels.

The PTGS2 gene encodes COX-2, an inducible enzyme associated with a variety of physiological responses, such as inflammation (59). In normal conditions, COX-2 is not detected in the gastrointestinal tract or expressed at low levels (60). COX-2 has been identified as a crucial mediator of IRI. COX-2 expression increased after mesenteric IRI was associated with gut inflammation, injury, and impaired ansit (9). In addition, mice with COX-2 deficiency showed decreased damage after IRI (61). Consistent with our findings, COX-2 expression increased significantly in the IR group. Nar pretreatment reduced COX-2 expression, suggesting that Nar mitigated intestinal IRI-induced inflammation, at least partially, by suppressing COX-2 expression.

YAP, which functions as a co-activator of the Hippo pathway, regulates the proliferation and differentiation of intestinal cells (62). The PPI network (**Figure 7A**) downloaded from STRING database indicated the interaction between YAP, STAT3, PTGS2, IL6, TNF, IL1B, and IL10. Our study found that YAP was down-regulated significantly in intestinal IRI, and Nar pretreatment activated YAP and increased YAP nuclear translocation levels, which aligns with our previous study (19). YAP’s role was further examined in the Nar’s anti-inflammatory effects against intestinal IRI. Intestinal IRI induced upregulation of PTGS2, p-STAT3, and pro-inflammatory cytokines, which was reversed by Nar. When VP inhibited YAP expression, Nar failed to reduce PTGS2, p-STAT3 and proinflammatory cytokines. Thus, Nar could activate the YAP pathway to inhibit STAT3 activation, reduce inflammation, ultimately alleviating intestinal IRI, as was consistent with previous studies. Some studies found that YAP suppressed inflammation progression. In bacterial pneumonia, type II alveolar epithelial cells needed YAP to activate IκBa in order to reduce nuclear factor (NF)-κB-mediated inflammation and promote recovery after pneumonia (63). In osteoarthritis, YAP suppressed NF-κB signaling and promoted cartilage repair (64). In IBD, YAP inhibited colitis inflammation and enhanced intestinal epithelial barrier repair by inhibiting jumonji domain-containing protein 3 by binding to enhancer of zeste homolog 2 (65). However, some studies also showed that YAP had pro-inflammatory functions. In IBD, YAP inhibited M2 macrophage polarization, whereas it stimulated IL-6 production by M1 macrophages activated by LPS/interferon γ (IFN-γ) (66). In human umbilical vein endothelial cells, naringin inhibited Ox-LDL-triggered apoptosis and inflammatory cytokines by inhibiting YAP (67). YAP plays a complex role in the inflammatory process, depending on the type of cell and microenvironment (68).

Many Studies have highlighted YAP as the targets of natural compounds in various diseases. One study found that celastrol effectively mitigated ferroptosis and intestinal IRI-induced acute lung injury by increasing YAP levels (69), which is consistent with our study suggesting that Nar exerts anti-inflammatory effects by activating YAP in intestinal IRI. Furthermore, we previously demonstrated a strong affinity between Nar and YAP through molecular docking analysis and molecular dynamics simulations. In contrast, another study found that asiaticoside treatment reduced the malignancy of breast cancer cells by diminishing YAP expression (70). By downregulating YAP, colosolic acid inhibited lung cancer cell metastasis (71). Differences in the disease models may account for this discrepancy. For this reason, further research into how Nar regulates YAP protein levels is needed. STAT3 is reported to be a novel transcriptional factor partner of YAP, mediating YAP’s proangiogenic effects (72). In our previous study, Co-IP and immunofluorescence results confirmed the interaction between YAP and STAT3 in IEC-6 cells, which is consistent with one study suggesting that YAP interacted with STAT3 to promote macrophage M2-type polarization induced by breast cancer cell supernatant (73).

Our study suggests that Nar can alleviate intestinal IRI-induced inflammation and exert protective effects through the YAP/STAT3 pathway. The following limitations exist in our study. First, the data sources for the network pharmacology analysis are derived solely from databases, whereas we didn’t perform omics analyses of animal tissues before and after Nar administration. Second, the mechanisms by which Nar regulates immune cell infiltration and macrophage polarization in intestinal IRI remain poorly understood. Further experiments are needed to elucidate these processes. Third, to regulate YAP levels, neither genetic knockout mice nor lentiviral transfections were used. Finally, biomarkers for the assessment of intestinal permeability are lacking in our study. It is urgently necessary to address these limitations in the future to ensure scientific and reliable results.

## 5 Conclusion

To summarize, employing network pharmacology in combination with experimental assays, we demonstrated that Nar pretreatment improved intestinal IRI-induced inflammatory responses by activating YAP signaling to negatively regulate STAT3 phosphorylation. The results indicate that Nar may prove to be an effective therapeutic agent for intestinal IRI, but further experiments are still needed to explore its mechanism.

## Data Availability

The original contributions presented in the study are included in the article/supplementary material. Further inquiries can be directed to the corresponding author/s.
